# Winter-Spring dynamics of dissolved organic carbon fluxes driven by precipitation in a North Carolina tidal marsh

**DOI:** 10.1016/j.ecss.2025.109361

**Published:** 2025-09-15

**Authors:** Marie Cindy Lebrasse, Blake A. Schaeffer, DelWayne R. Bohnenstiehl, Christopher L. Osburn, Megan M. Coffer, Ruoying He, Peter J. Whitman, Wilson B. Salls, David D. Graybill

**Affiliations:** a Department of Marine, Earth and Atmospheric Sciences, North Carolina State University, Raleigh, NC, USA; b U.S. Environmental Protection Agency, Office of Research and Development, USA; c Now at Silvestrum Climate Associates, B.V., Jisp, The Netherlands; d Center for Geospatial Analytics, North Carolina State University, Raleigh, NC, USA; e Planet Labs, San Francisco, CA, USA; f United States Geological Survey, NY, USA; g AECOM, Wilmington, NC, USA

**Keywords:** Blue carbon, Tidal marshes, Dissolved organic carbon, Numerical modeling

## Abstract

The lateral transport of dissolved organic carbon (DOC) is crucial in tidal marsh carbon budgets, but estimating DOC remains a challenge due to the dynamic nature of terrestrial-aquatic interfaces and limitations of *in situ* observations. This study used a linear model to correlate DOC and chromophoric dissolved organic matter (CDOM) absorption coefficient at 440 nm ($a_{\text{CDOM}}(440)$; $r^{2}=0.75$) in Bald Head Creek, North Carolina, USA. Modeled DOC concentrations were merged with current velocity derived from the Semi-implicit Cross-scale Hydroscience Integrated System Model (SCHISM) to estimate daily DOC fluxes from January to April 2021, representing winter and spring in the mid-latitude northern hemisphere. DOC flux depended on season and tide, where negative values represent DOC import into the marsh and positive values represent export to the estuary. Results showed that DOC was exported from the marsh in February and March after peak rainfall. While tidal inundation and increased river flow from precipitation influenced DOC flux, the average daily DOC import of −13.6 ± 6.0 g C m^−2^ d^−1^ was greater than the average daily DOC export of 9.4 ± 8.3 g C m^−2^ d^−1^ during the study period, showing that the marsh was a net sink for DOC during the early spring. This study demonstrated that field measurements with models like SCHISM provided a synoptic representation of DOC flux, where rainfall-induced river discharge impacts DOC mobilization and export to coastal systems like Bald Head Creek.

## 1. Introduction

Tidal salt marshes are networks of dendritic, finger-like intertidal habitats that occur mostly along the coastline at mid-to high-latitudes. Tidal salt marshes facilitate movement of tidal water onto the marsh surface at high tide and back into adjacent coastal waters at low tide (Sanger and Parker, 2016). These terrestrial-aquatic interfaces are biogeochemically dynamic ecosystems that receive and rapidly process organic matter and nutrients delivered from their adjacent landscape and seaward boundary (Fogel et al., 1989; Boschker et al., 1999; Jiménez-Ramos et al., 2025). Covering an estimated area of 18,510 km^2^ in the United States alone (Worthington et al., 2024), tidal marshes are hotspots for carbon cycling due to their unique biogeochemical and hydrogeomorphic processes, which are sensitive to hydrodynamics and complex circulation patterns forced by tides, winds and river flow (Tzortziou et al., 2008; Fagherazzi et al., 2013; Osburn et al., 2015; Clark et al., 2018, 2020; Bogard et al., 2020; Taylor et al., 2021).

Anthropogenic carbon dioxide (CO_2_) emissions absorbed from the atmosphere and stored by vegetated coastal ecosystems (*e.g*., seagrasses, tidal marshes, mangroves) are termed blue carbon. Tidal marshes accumulate organic carbon in their soils at a rate ~55 times faster than tropical rainforests and store this carbon for millennia (McLeod et al., 2011) constituting one of the largest blue carbon sinks. The potential global carbon stocks in the top metre of tidal marsh soil is estimated at 1.22 ± 0.20 Pg C (Maxwell et al., 2023). Yet, the sustainability of this long-term blue carbon storage is threatened by environmental disturbances linked to both human-driven changes in land use and global climate change (Thorne et al., 2015; Gilby et al., 2021). Land use changes affect the supply of freshwater, nutrients and sediments from coastal watersheds to tidal marshes, influencing carbon cycling (Weston et al., 2014; Altieri and Gedan, 2015; Colombano et al., 2021; Gilby et al., 2021). Blue carbon loss from tidal marshes has already been reported due to land reclamation (Bu et al., 2015; Lewis et al., 2019), eutrophication (Deegan et al., 2012), and chemical and physical disturbances (Mason et al., 2003; Greenberg et al., 2006; Macreadie et al., 2013). Further, accelerating rates of sea-level rise (Watson et al., 2015; Nerem et al., 2018; Dangendorf et al., 2019; Holmquist et al., 2021; Saintilan et al., 2022) may worsen the effect of *coastal squeeze*, a process whereby coastal habitats such as tidal marshes are caught between rising sea levels and human-made structures like seawalls, dikes, or other coastal defenses (Doody, 2013). Through this process, marshes may be unable to migrate landward due to high water being fixed by anthropogenic and geomorphic barriers and increased coastal erosion (Crosby et al., 2016; Leo et al., 2019; Colombano et al., 2021; Gilby et al., 2021). Ultimately, the loss of habitat from these disturbances can result in blue carbon loss from tidal marshes through greenhouse gas emissions back to the atmosphere (Beckett et al., 2016; Himes-Cornell et al., 2018). While protecting and maintaining tidal marshes to offset greenhouse gas emissions is a high priority for climate change mitigation efforts, quantification of carbon stocks and processes that control carbon cycling is also important (Macreadie et al., 2017).

Since Teal’s (1962) studies on energy flow through a salt marsh ecosystem, there has been continued interest in the contribution of tidal marshes to the carbon budget of coastal ecosystems. The two primary forms of organic carbon that are exchanged between tidal marshes and their adjacent estuarine and coastal ecosystems are dissolved and particulate organic carbon (DOC and POC, respectively). Our study focuses on DOC, defined as the organic carbon component of matter that can pass through a filter, typically between 0.70 and 0.22 μm in size. DOC is a major component of aquatic ecosystems, where oceanic DOC is comparable to carbon stored in the atmosphere (Jiao et al., 2010; Thornton, 2014), and it constitutes an estimated 80–90 % of the total organic carbon in the coastal ocean (Hedges, 1992). Tidal marshes act as important mediators of DOC storage and transport between upland terrestrial and river sources and the offshore marine environment. The sources of DOC in upland and marine environments can vary drastically in composition, where upland sources are likely derived from coastal vegetation, atmospheric deposition, river inputs, and groundwater discharge; and marine sources are likely derived from phytoplankton exudates, zooplankton decomposition, and benthic fluxes. DOC plays a key role in the biogeochemistry of tidal marshes (Tobias and Neubauer, 2019), where it forms the foundational energy sources for the microbial community.

The spatial and temporal variability in DOC can be quantified using its light-absorbing fraction, termed chromophoric dissolved organic matter (CDOM). Higher CDOM concentration is found in coastal regions where riverine and terrestrial inputs dominate, while lower CDOM concentration is found in open ocean environments (Coble, 2007). CDOM and DOC are often highly correlated in coastal systems with a salinity gradient such as tidal marshes, due to mixing of terrestrial, oceanic, and marsh-derived sources (Tzortziou et al., 2008). With the ability to obtain *in-situ* CDOM measurements at high frequency using fluorometers, CDOM has been extensively used as a proxy for DOC in such systems and helps quantify DOC export from terrestrial to marine environments (Asmala et al., 2012; Fichot and Benner, 2012; Vantrepotte et al., 2015; Osburn et al., 2016; Massicotte et al., 2017; Cao et al., 2018).

The lateral transport of DOC to adjacent ecosystems has been increasingly recognized as an important component of carbon budgets in tidal marshes, but estimates are variable, ranging from approximately 20 to 328 g C m^2^ yr^−1^ (*e.g*., Tobias and Neubauer, 2019; Osburn et al., 2015; Herrmann et al., 2015; Najjar et al., 2018; Schiebel et al., 2018). In tidal marshes, DOC is often exchanged at very small spatial and temporal scales and over short time periods. Thus, these marshes are often underrepresented in global and coastal carbon budgets due to the difficulty in observing, measuring, and modeling carbon fluxes in such spatially compressed and temporally dynamic zones. According to the second *State of the Carbon Cycle Report*, the link between tidal marsh systems and their adjacent estuaries is still an area of the coastal ocean carbon cycle with large uncertainties despite decades of research (Windham-Myers et al., 2018). Along with these uncertainties, the drivers of tidal and seasonal variations in DOC concentrations and fluxes remain poorly quantified in tidal marshes (Bauer et al., 2013; Benway et al., 2016; Najjar et al., 2018; Windham-Myers et al., 2018). A challenge in synthesizing flux rates across studies is a lack of carbon mass attributed to a defined wetland area, which can be difficult due to complicated surface and subsurface hydrologic flows (Menendez et al., 2022). Among other factors, differences in sampling duration and frequency and geographic location may contribute to the difficulties in accurately quantifying carbon budgets in tidal marsh systems (Knobloch et al., 2021). Additionally, there could be large uncertainties in net lateral carbon fluxes because of temporal variability, such as episodic events not captured during the period of flux calculation (Najjar et al., 2018). Thus, there is a clear need to better constrain the dominant physical and biogeochemical controls on these fluxes and create more accurate coastal carbon budgets.

Here, we combined *in situ* CDOM data with numerical model results to estimate DOC fluxes from a tidal creek (Bald Head Creek; BHC) draining a small, estuarine-fronting tidal marsh in southeastern North Carolina, USA. This study leveraged a strong correlation between *in situ* CDOM absorption at 440 nm ($a_{\text{CDOM}}(440)$) and DOC in combination with water flux derived from the Semi-implicit Cross-scale Hydroscience Integrated System Model (SCHISM) to estimate lateral DOC flux from marsh to estuary. We posed the following research questions: **(1)** What is the quantitative relationship that exists between $a_{\text{CDOM}}(440)$ and DOC measured at discrete, georeferenced locations? **(2)** Does the lateral DOC flux estimated from *in situ* DOC combined with model-derived water flux exhibit any seasonal pattern? This approach of combining *in situ* measurements and hydrodynamic modeling to estimate lateral DOC flux can be beneficial for determining the linkages between physical processes and transport of carbon in small coastal areas (Lehrter et al., 2013).

## 2. Methods

### 2.1. Study area

**Fig. 1.**
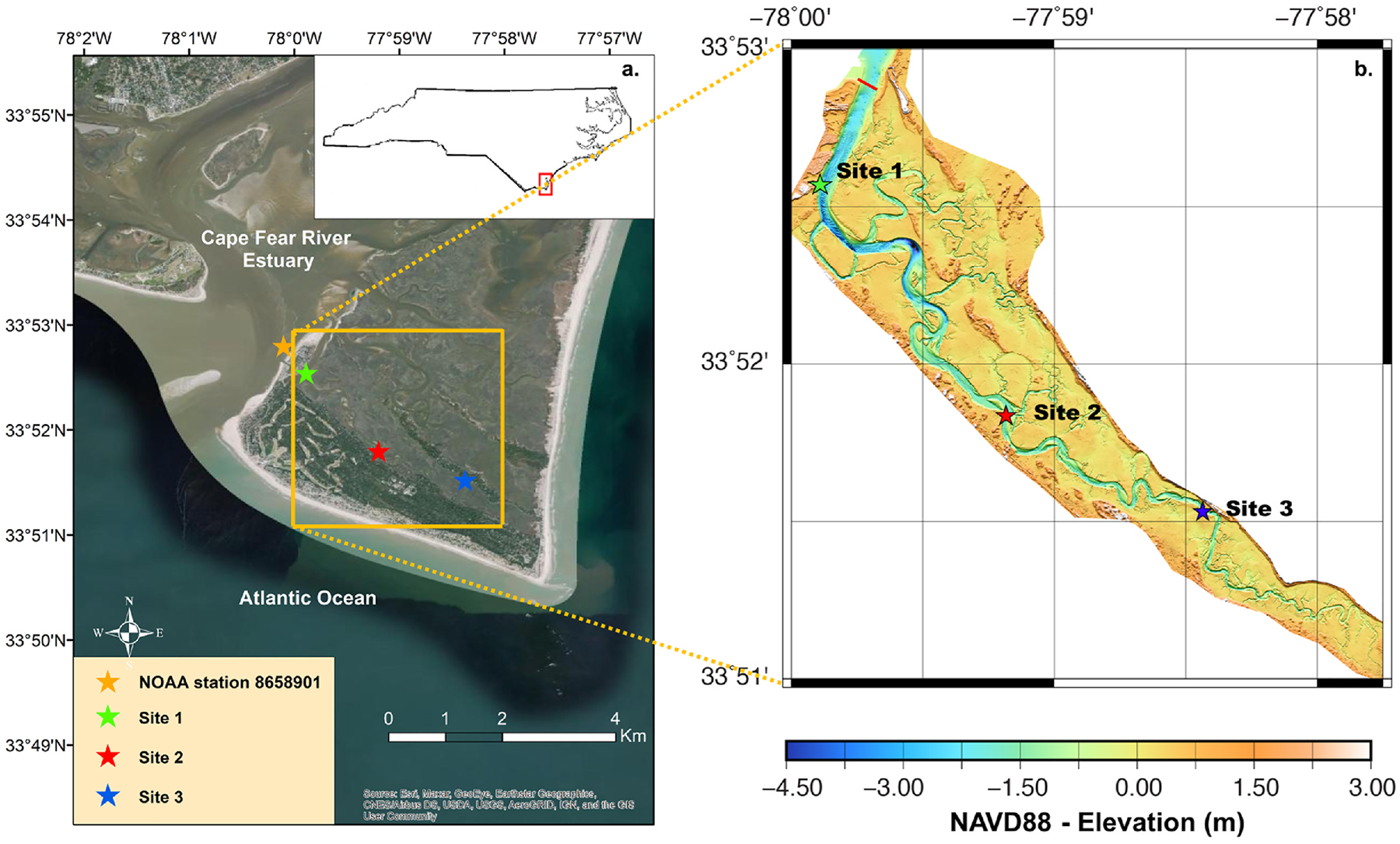
(a) Map of study site at Bald Head Creek in North Carolina, USA, showing locations of the three sampling sites and the NOAA water level station 8658901 and the Cape Fear River Estuary. (b) The digital elevation model (DEM) generated from high-resolution bathymetric mapping of the tidal creek channel and NOAA bathymetric lidar (Office for Coastal Management, 2018) to model Bald Head Creek using the numerical model SCHISM. The red line close to the mouth of the creek represents the cross-section drawn for DOC flux and export calculations.

Home to a large nature preserve, Bald Head Island is a 10 km^2^ barrier island located at the mouth of the Cape Fear River, North Carolina, USA (33.86°N, 77.99°W) and is bounded by the Atlantic Ocean to the south and east and by tidal salt marshes to the north (Sherrill et al., 2010) (Fig. 1a). Bald Head Creek is a pristine tidal creek on Bald Head Island, that drains approximately 4.2 km^2^ of productive salt marshes into the estuarine Cape Fear River, west of the island. The tidal creek receives brackish water from North Carolina’s largest and most industrialized watershed via the Cape Fear River and two black-water tributaries, the Black and Northeast Cape Fear Rivers, which drain bottomland hardwood forested swamps high in organic matter (Becker et al., 2010). The Cape Fear River estuary (Fig. 1a) is characterized by a relatively large, open mouth, which allows tidal currents to propagate well into the system and its adjacent creeks such as BHC. The vegetation on the marsh platform is mostly dominated by saltmarsh cordgrass (*Spartina alterniflora*) and black needlerush (*Juncus roemerianus*), with a transitional fringe of saltgrass (*Distichlis spicata*), sea ox-eye (*Borrichia frutescens*) and seacoast marshelder (*Iva imbricate*).

Elevations of the marsh platform range from about 0 to 0.5 m according to the North American Vertical Datum of 1988 (NAVD88; Fig. 1b). Tides in the estuary are semi-diurnal, with the mean of the daily lowest tides at −0.91 m and the mean of the daily highest tides at 0.61 m (National Oceanic and Atmospheric Administration (NOAA) tides and currents, station ID 8658901, Fig. 1a). Whole water samples from three sites along BHC have been collected by the Bald Head Island Conservancy (BHIC) since 2014 for water quality monitoring purposes. Site 1 (33.8757°N, 77.9981°W) is located near the mouth of the creek where the Cape Fear River estuary converges with the Atlantic Ocean (Long Bay) near the village of Bald Head Island. Site 2 is in the middle of the creek (33.8633°N, 77.9866°W), and Site 3 is located near the head of the creek (33.8587°N, 77.9729°W).

### 2.2. Water sampling and field sensor deployment

#### 2.2.1. Sample collection and processing and optical analysis

Fourteen water samples were collected by BHIC between January and April 2021 (Table 1). These samples were collected in cleaned (detergent-washed and thoroughly rinsed with Milli-Q water) 1000 ml brown high-density polyethylene bottles and stored on ice for processing. In the laboratory, samples were filtered through pre-combusted (450 °C for 5 h) and pre-rinsed (150 ml Milli-Q water) Whatman GF/F filters (pore size 0.7 μm) using gentle vacuum within 24 h of sample collection. The filtrate was divided into a 60-ml polycarbonate bottle for CDOM absorption and a 40-ml borosilicate glass vial (detergent-washed and thoroughly rinsed with Milli-Q water, then baked at 450 °C for 5 h) for DOC measures (See section 2.2.2 for DOC analysis details). The CDOM samples were kept refrigerated at 4 °C until analysis.

CDOM absorbance on the filtered samples was measured on a Varian Cary 300UV spectrophotometer in 1 cm quartz cells at wavelengths ranging from 200 to 800 nm and blank corrected with Milli-Q water. Napierian absorption coefficients ($a_{\text{CDOM}}$) were computed in units of m^−1^ using the following equation following Osburn and Morris (2003):

(1) $$a_{\text{CDOM}}\lambda =\frac{A\left(\lambda_{\text{sample}}\right)-A\left(\lambda_{\text{blank}}\right)}{L}\times 2.303$$

where $a_{\text{CDOM}}$ is the absorption coefficient, λ is the wavelength in nanometers, A is the absorbance (optical density) of the sample or blank measured at wavelength λ on the spectrophotometer, L is the pathlength of the quartz cell in meters, and the factor 2.303 converts from a log_10_ to the natural logarithm. $a_{\text{CDOM}}(440{)}_{\text{insitu}}$ was then used to quantify CDOM since this wavelength corresponds to the blue wavelength where CDOM dominates light absorption (Del Vecchio and Subramaniam, 2004; Pan et al., 2008; Pahlevan et al., 2019; Hooker et al., 2020; Houskeeper et al., 2021).

#### 2.2.2. DOC analysis

DOC analysis was conducted on a modified OI Analytical Aurora Model 1030 Total Organic Carbon analyzer, using a high temperature combustion method (Lalonde et al., 2014). After filtration and prior to analysis, the samples were acidified to pH 2 with 85 % phosphoric acid (Tisserand et al., 2024) and sparged using ultra-high purity Argon gas for 20 min to remove any dissolved inorganic carbon. DOC values (mg C L^−1^) were blank-corrected against Milli-Q water and calibrated against six caffeine standards, ranging from 1 to 40 mg C L^−1^, and two sucrose standards ranging from 2 to 5 mg C L^−1^ obtained from the International Atomic Energy Agency (IAEA C6). The 14 water samples collected during the winter-spring of 2021 (January to April) in BHC were termed ${\text{DOC}}_{\text{insitu}}$. The relationship between $a_{\text{CDOM}}(440{)}_{\text{insitu}}$ and ${\text{DOC}}_{\text{insitu}}$ was fit using a Model II linear regression.

#### 2.2.3. Field sensor deployment

Site 2 was instrumented with a water level meter (HOBO bottom pressure logger, accuracy ±3 mm, Onset Computer Corporation, Bourne, MA, USA), a tilt-style current meter (Lowell TCM-4 Shallow Water Tilt Current Meter; ±2 cm s^−1^, Lowell Instruments, LLC) and a fluorescent DOM (fDOM) sensor on an EXO3 multiparameter sonde (YSI Inc., USA) over 4 weeks in the summer of 2021 (17 June – 14 July 2021). Because the purpose of this field data collection was to calibrate the numerical model, this time period does not align with the study period. Shallow water level measurements were processed to account for the local atmospheric pressure changes using weather station observations (station BALD; https://econet.climate.ncsu.edu/stations/?id=BHIC#tab_current) available on Bald Head Island.

Sensors were deployed at Site 2 and configured to collect measurements every 5 min over a four-week period. Maintenance on the three instruments and data download were performed once during the four-week period to address biofouling. fDOM measured by the EXO3 sonde was converted to $a_{\text{CDOM}}(440)$ based on a linear calibration developed in the laboratory ${(r}^{2}=0.99).\;a_{\text{CDOM}}(440)$ derived from the EXO3 sonde is referred to as $a_{\text{CDOM}}(440{)}_{\text{EXO}}$ throughout the manuscript. The $a_{\text{CDOM}}(440{)}_{\text{EXO}}$ data was filtered for single outlier events in the time series, which exceeded three standard deviations above the mean or if there was a negative value. These outliers were preceded and followed with measures that fell within one standard deviation of the mean, suggesting it was an isolated spike.

### 2.3. Numerical model description

The Semi-implicit Cross-scale Hydroscience Integrated System Model (SCHISM) was used to simulate water current velocity in BHC so we could quantify DOC fluxes in the creek on each of the 14 sampling days. SCHISM is an open-source, 3D community model based on unstructured grids for hydrodynamics and ecosystem dynamics (Zhang et al., 2016). SCHISM uses a highly efficient and accurate semi-implicit finite-element/finite-volume method with the Eulerian-Lagrangian algorithm to solve the Reynolds-Averaged Navier-Stokes equations (Equation (2) through 5) to address a wide range of physical and biological processes. The semi-implicit method integrated in SCHISM allows the stable integration of the transport equations with a much larger time step than would otherwise be possible, hence reducing computation time. Standard model outputs from numerical models like SCHISM include water levels, salinity, temperature, and horizontal velocity.

Momentum equation:

(2) $$\frac{\partial u}{\partial t}=\frac{\partial }{\partial z}\left(v\frac{\partial u}{\partial z}\right)-g\nabla \eta +F$$

Continuity equation:

(3) $$\frac{\partial \eta }{\partial t}+\nabla .\int_{-h}^{\eta }u\partial z=0$$

(4) $$\nabla .u+\frac{\partial w}{\partial z}=0$$

Transport equation:

(5) $$\frac{\partial C}{\partial t}+\nabla .(uC)=\frac{\partial }{\partial z}\left(\kappa \frac{\partial C}{\partial z}\right)+F_{h}$$

where z is the vertical coordinate (upward), t is the time, η is the free surface elevation, h is the bathymetric depth, u represents the horizontal velocity with cartesian coordinates (u, v), w is the vertical velocity, F is the other forcing terms in momentum such as horizontal viscosity, Coriolis, Earth tidal potential and atmospheric pressure, g is the acceleration of gravity, C is the tracer concentration such as salinity or temperature, v is the vertical eddy viscosity, κ is the vertical eddy diffusivity for tracers, and $F_{h}$ is the horizontal diffusion. Together, these equations govern the SCHISM model and were used to resolve the hydrodynamics of BHC.

#### 2.3.1. Model set up, initialization and run

SCHISM uses an unstructured grid capable of resolving the complex geometry of tidal creeks. The grid was generated in the Surface-water Modeling System (SMS, Aquaveo, LLC, Provo, UT). The resulting horizontal model grid consisted of 94,482 triangular elements and 47,811 nodes. SCHISM also requires the following input variables: bathymetry interpolated to the model grid and open boundary conditions including water level and river discharge. Bathymetry of BHC was obtained as a 1 × 1 m resolution digital elevation model (DEM) of the creek and marsh platform based on bathymetric data collected in August 2016 using an Edgetech 6205 sonar mounted on an Uncrewed Surface Vehicle (system described in Caretti et al., 2021), and bathymetric light detection and ranging (LiDAR) data collected at low tide in the fall of 2014 following Hurricane Sandy (Office for Coastal Management, 2018) (Fig. 1b). Model bathymetry was subsequently interpolated from the 1-m resolution DEM to the model grid.

Time series of water level (from NOAA tides and currents, station ID 8658901 on Bald Head Island, last accessed Sep 2021) and Cape Fear River discharge data (US Geological Survey’s station 02105769 at Kelly, NC; waterdata.usgs.gov) were used as open boundary conditions in the model. Water level was defined relative to the same NAVD88 datum as the model bathymetry. The high and low tides as well as the 15-min river discharge data for a period spanning November 2020 to November 2021 were downloaded and interpolated to data at 5-min intervals via shape-preserving piecewise cubic interpolation (Mathworks, Inc) to match the 5-min frequency at which SCHISM model simulations were performed. Table 2 describes the distribution of each input variable, which can provide a reference for broader model applicability.

The numerical model simulation was conducted for a 19-day period spanning 25 June 2021 00:00 UTC to 13 July 2021 23:59 UTC, including a two-day spin-up to allow for hydrodynamic adjustment in the model for testing and validation. Then the model was run for the period spanning January 00:00 UTC to April 2021 23:59 UTC to obtain water flux measurements for each of the sampling dates. The dot product result between the unit-normalized modeled and observed current velocity was also computed to determine if both datasets agree in direction. A dot product result of 1 means that the modeled and observed current velocity go in the same direction as each other; a dot product result of 0 means that the modeled current velocity direction is at 90° (orthogonal) to the observed current velocity direction. A dot product result of −1 means that the modeled and observed current velocity go in the opposite direction to each other.

#### 2.3.2. Quantification of lateral DOC fluxes

${\text{DOC}}_{\text{insitu}}$ derived from 14 water samples collected during the winter-spring of 2021 (January to April) in BHC were merged with water flux modeled in SCHISM for the creek during each water sampling period to quantify DOC fluxes.

Water flux (Q, m^3^s^−1^) was computed as the product of the along-channel averaged velocity and the cross-sectional area of the channel (Equation (6); Ganju et al., 2005; Downing et al., 2009). The reference surface for these calculations was a vertical cross section oriented locally normal to the channel (azimuth 112°) with a width defined by the upper margins of the channel banks (profile length 147 m). Water levels and current velocity data from the model output were interpreted at points every 0.5 m along the cross-sectional profile. The heights along the flooded cross-section (h_i_) were calculated by differencing the water level data (at each time, t) with the bathymetry:

(6) $$Q(t)=\sum_{i=1}^{n}0.5h_{i}\left[u_{i}(t),v_{i}(t)\right]\cdot N$$

where n is a unit normal vector to the cross section oriented downstream and the summation is over each point, N, along the cross section. Lateral DOC flux (g C s^−1^) for each water sampling date was calculated by multiplying the instantaneous water flux (m^3^ s^−1^) by the DOC (g m^−3^) at the cross section near the mouth of the creek (Site 1). Daily lateral DOC export measurements were then computed by dividing DOC flux by the maximum flooded marsh surface area. Daily lateral DOC export was reported per unit area in g C m^−2^ d^−1^. The lateral DOC flux was compared to the excess rainfall hyetograph for Bald Head Island. Excess rainfall, which is the volume of rainfall available for direct surface runoff and which contributes to the streamflow at the watershed outlet, is computed by applying the Soil Conservation Service’s Curve Number method (Mishra and Singh, 2003) to rainfall data from NC State Climate Office’s station BALD on Bald Head Island.

## 3. Results

### 3.1. In situ data

**Fig. 2.**
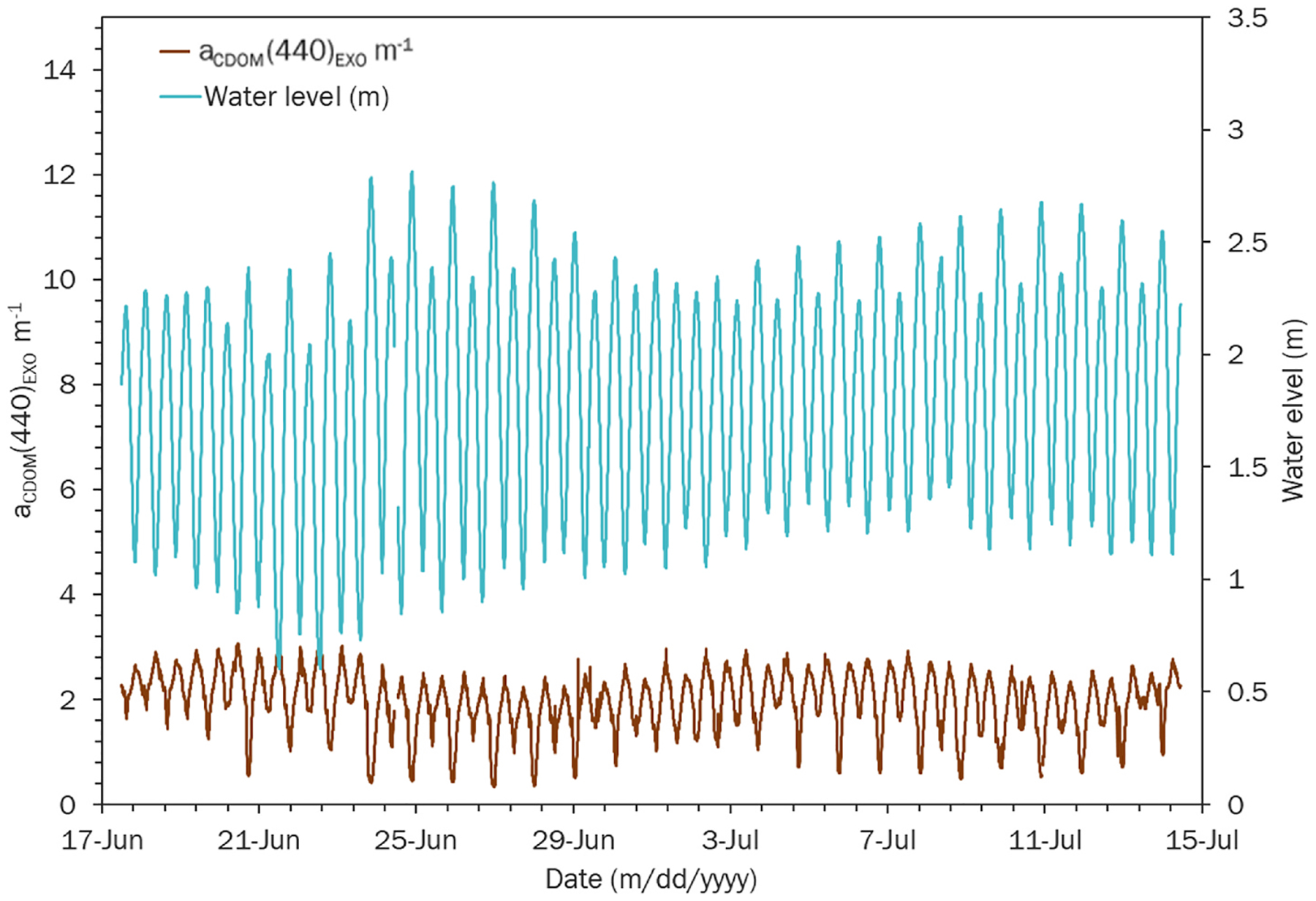
Time series of $a_{\text{CDOM}}(440{)}_{\text{EXO}}$ measured by the EXO3 multiparameter sonde and water level measured by the HOBO bottom pressure logger during the 17 June through 14 July 2021 field deployment period.

Measurements of $a_{\text{CDOM}}(440{)}_{\text{EXO}}$ at Site 2 ranged between a minimum of 0.34 m^−1^ on 26 June at 11:40 a.m. Eastern Daylight Time (EDT; water level = 2.73 m) and a maximum of 3.09 m^−1^ on 22 June at 13:40 p. m. EDT (water level = 0.65 m) (Fig. 2). The $a_{\text{CDOM}}(440{)}_{\text{EXO}}$ measurements averaged 1.96 ± 0.59 m^−1^ over the 4-week period. Tidal flux of $a_{\text{CDOM}}(440{)}_{\text{EXO}}$ was evident throughout the 4-week period where an opposite pattern was observed between $a_{\text{CDOM}}(440{)}_{\text{EXO}}$ and water level. Higher $a_{\text{CDOM}}(440{)}_{\text{EXO}}$ values were observed at low tide, gradually decreasing as water flowed into the creek during flood tide and reaching minimum concentrations at high tide (Fig. 2). As water drained off the marsh platform of BHC at ebb tide, $a_{\text{CDOM}}(440{)}_{\text{EXO}}$ values gradually increased again.

### 3.2. $a_{\text{CDOM}}(440{)}_{\text{insitu}}$ to DOC relationships

**Fig. 3.**
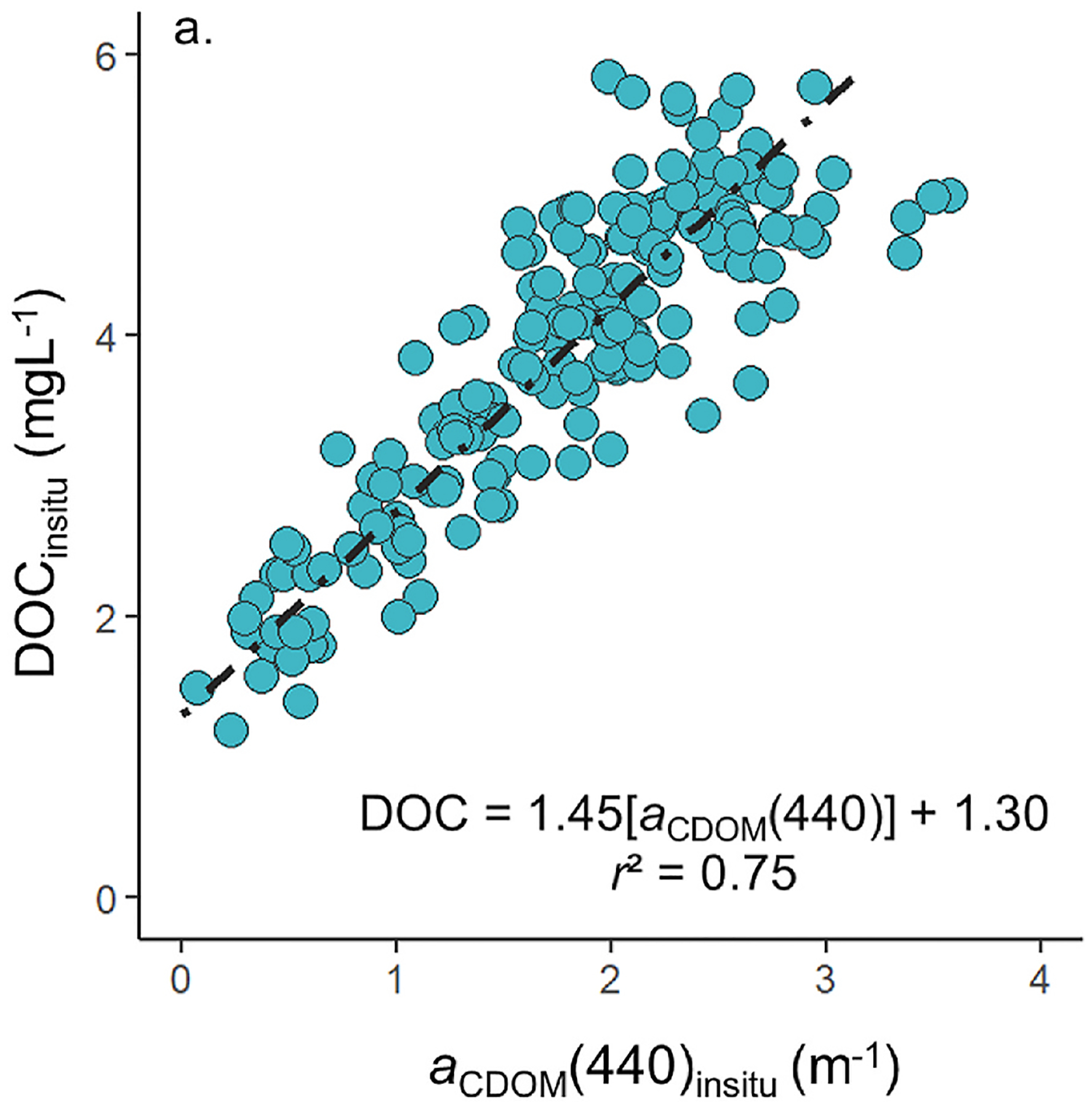
Linear regression calibration between $a_{\text{CDOM}}(440{)}_{\text{insitu}}$ and ${\text{DOC}}_{\text{insitu}}$ collected between 2016 and 2020 (n = 186) in Bald Head Creek, NC.

A relationship between $a_{\text{CDOM}}(440{)}_{\text{insitu}}$ and ${\text{DOC}}_{\text{insitu}}$ was developed from *in-situ* data collected between 2016 and 2020 in BHC, which showed a strong correlation between both parameters ($r^{2}=0.75,\;p<0.001$; Fig. 3; Equation (7)). This relationship was then applied to the 14 $a_{\text{CDOM}}(440{)}_{\text{insitu}}$ measures spanning January to April 2021 to derive the DOC values merged with modeled-derived current velocity to compute the daily DOC flux.


(7) $${\text{DOC}}_{\text{insitu}}=1.45\times a_{\text{CDOM}}(440{)}_{\text{insitu}}+1.30$$

### 3.3. SCHISM model validation

**Fig. 4.**
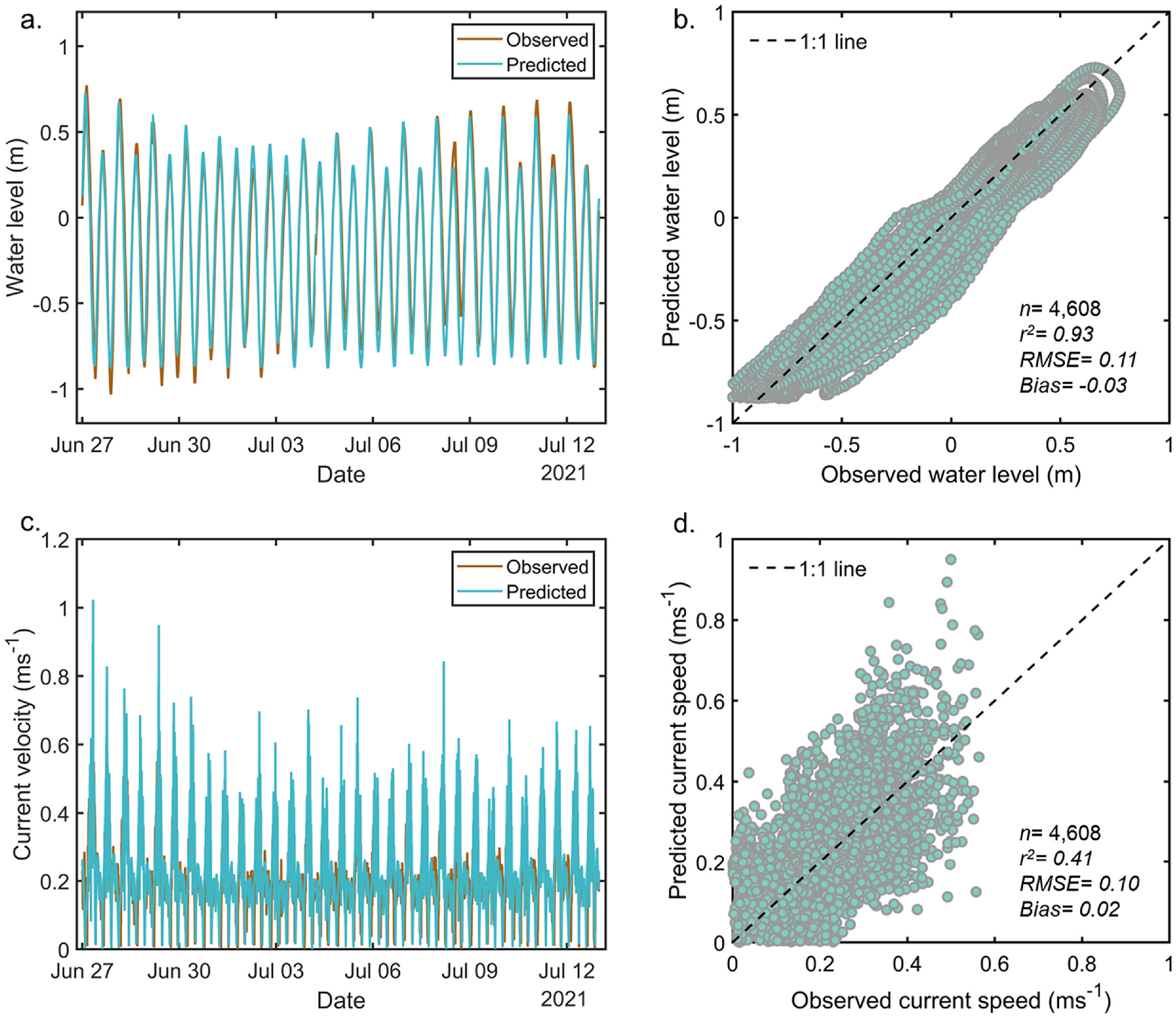
Time series and match-up comparisons between observed and predicted (a–b) water level, and (c–d) current velocity.

A time series of modeled water level for a 19-day period spanning 25 June 2021 to 13 July 2021 was compared to water level collected at Site 2 within the creek during the same 19-day period (Fig. 4a). The time series showed that the SCHISM model accurately reproduced the variations in the observed water level. A validation between modeled and observed water levels showed excellent agreement ($r^{2}=0.93$), with the 4608 values closely distributed along the 1:1 line (Fig. 4b). Based on the model assessment metrics, the model showed satisfactory performance in simulating water level at Site 2 with a low root mean square error (RMSE) (0.11 m) and low bias (−0.03 m). Similarly, modeled current velocity extracted at the Site 2 coordinates was compared to current velocity collected at Site 2 in the creek for the 19-day period. The time series showed that the model reproduced the pattern observed in current velocity at Site 2 (Fig. 4c). Validation between modeled and observed current velocity demonstrated satisfactory performance of the model with a RMSE = 0.10 ms^−1^ and bias = 0.02 ms^−1^ (Fig. 4d).

**Fig. 5.**
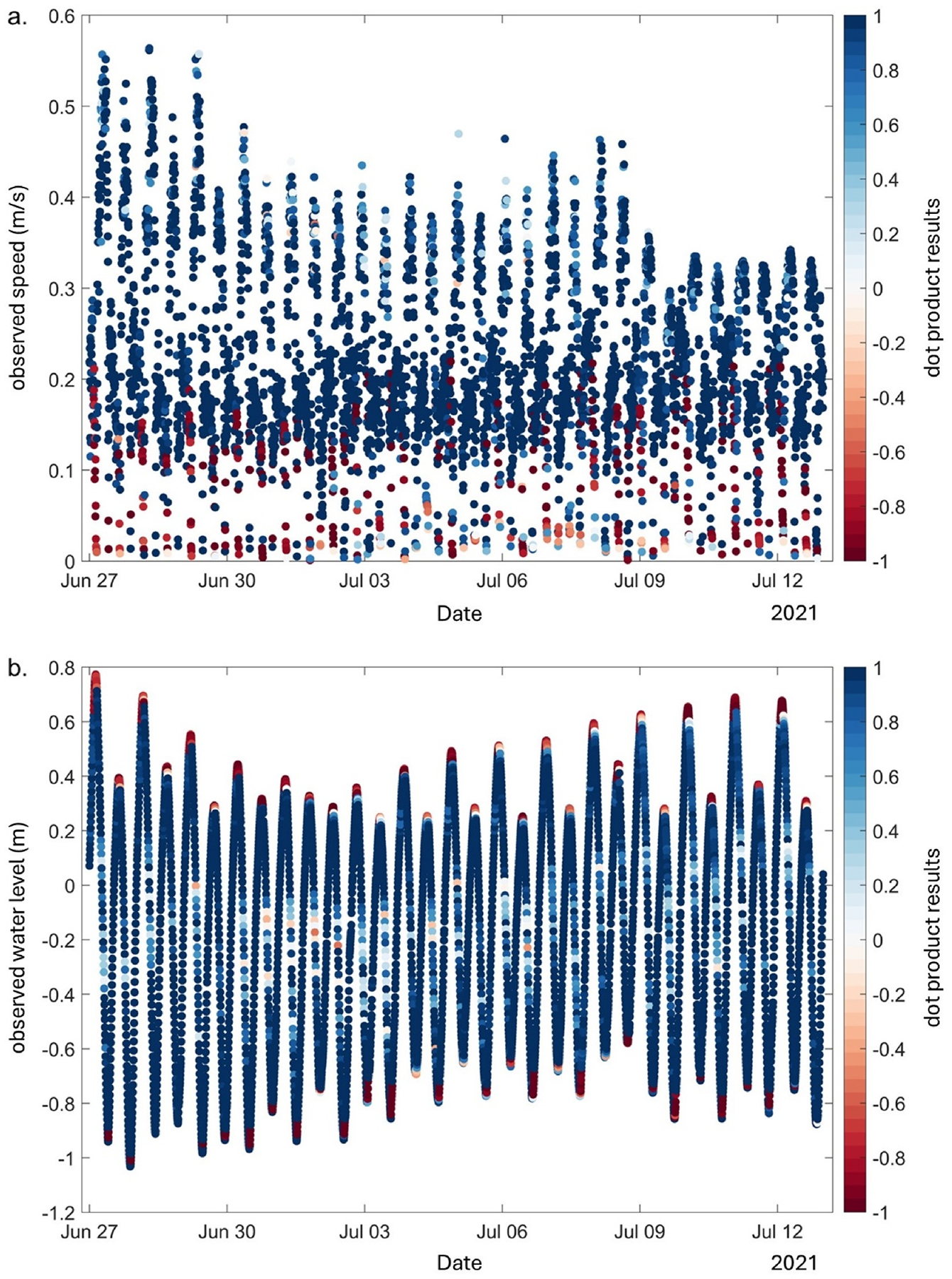
The dot product between observed (a) current speed and (b) waver levels measured *in situ* at Site 2 as a function of time. Colors indicate the dot product of the unit normalized observed and modeled current directions. A dot product of 1 means that SCHISM was able to reproduce the observed current direction, a dot product of 0 means that modeled current directions were at 90° with observed current directions and a dot product of −1 means that velocities were in opposite directions.

The dot product result between the unit-normalized modeled and observed current velocity was computed to determine if both datasets agree in direction (Fig. 5a). Out of the 4608 data points extracted from the 19-day model simulation (excluding the first 2 days as the model ramp-up period), 88 % had a dot product between 0.6 and 1 with the observed current velocity and 10.8 % had a dot product from −0.6 to −1. The data points that had a dot product from −0.6 to −1 had a current speed of 0–0.1 ms^−1^. Plotting the dot product along with the water level showed that the instances where the dot product of observed and modeled current velocity was −1 occurred at slack tide, where the tide was changing directions (Fig. 5b).

### 3.4. Lateral DOC flux and export from BHC

**Fig. 6.**
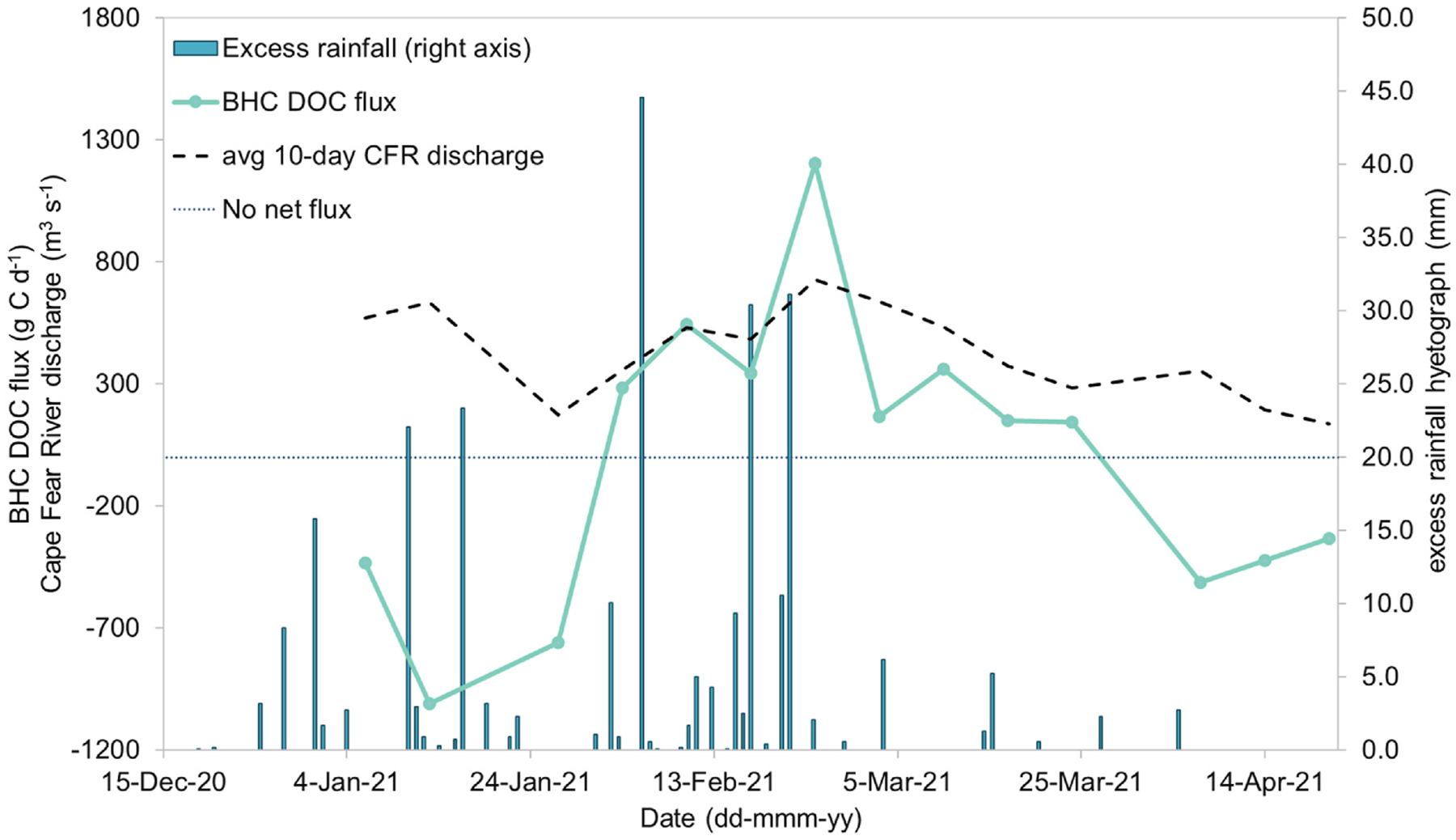
Average daily DOC flux for the months of January through April 2021. Negative values represent import to, or uptake by the marsh while positive values represent export from the marsh. Measurements were compared with the 10-day average river discharge (10 days prior to water sampling) from the USGS station 02105769 in the Cape Fear River and the excess rainfall hyetograph computed using rainfall data from the North Carolina State Climate Office’s station BALD on Bald Head Island.

Using the model-derived current velocity and the DOC values derived from Equation (7), daily lateral DOC flux from BHC was computed near the mouth of the creek (Site 1) for 14 dates in 2021, spanning January to April (Fig. 6; Table 3). Negative values represent import into, or uptake by, the marsh, while positive values represent export from the marsh. Substantial variability in flux was noted with respect to both import and export of DOC. For example, during the month of January 2021, an average flux of −17.3 ± 6.8 g C m^−2^ d^−1^ was observed, indicating import into the marsh-creek system, while for February, the average DOC export was 14.0 ± 9.9 g C m^−2^ d^−1^ and was notably coincident with the highest Cape Fear River discharge measured during the study period (Fig. 6; Table 3). In March, the average lateral DOC export was estimated at 4.84 ± 2.43 g C m^−2^ d^−1^ followed by an average lateral DOC import of −9.94 ± 2.10 g C m^−2^ d^−1^ in April. During late winter, when both river discharge and excess rainfall were at their highest, DOC export appeared to rise along with river discharge and excess rainfall, whereas this pattern was not evident when river discharge was low (Fig. 6).

## 4. Discussion

Tidal marshes play a critical role in both processing and exchanging DOC with adjacent estuaries and coastal waters (Ward et al., 2020). Thus, DOC dynamics at the estuarine-marsh interface provides an important link between terrestrial and aquatic carbon cycles. Here, we demonstrated a multiplatform approach for monitoring DOC dynamics in the estuarine-marsh interface of the BHC tidal marsh. The approach consisted of three major components: (i) A YSI fDOM sonde; (ii) DOC derived from $a_{\text{CDOM}}(440)$ based on the strong relationship between *in situ* $a_{\text{CDOM}}(440)$ and DOC; (iii) a numerical hydrodynamic model whereby SCHISM-modeled current velocity was used to estimate DOC flux from BHC.

### 4.1. Seasonality in $a_{\text{CDOM}}(440)$ and DOC dynamics

Several previous studies focused on DOC dynamics in coastal environments and terrestrial-aquatic interfaces were based on the seasonally and regionally specific relationships between absorption coefficient of CDOM and DOC (Mannino et al., 2008; Joshi et al., 2017; Cao et al., 2018; Chen et al., 2020; Cao and Tzortziou, 2021). Similarly, in BHC, the conservative relationship between $a_{\text{CDOM}}(440{)}_{\text{insitu}}$ and ${\text{DOC}}_{\text{insitu}}$ allowed for the development of an empirical algorithm to derive estimates of DOC from high frequency $a_{\text{CDOM}}(440{)}_{\text{EXO}}$ sonde observations.

Application of the CDOM empirical algorithm to DOC revealed a clear seasonal pattern in DOC distribution throughout the creek (Fig. 6). In general, values depicted the widely reported conservative behavior of CDOM and DOC along the salinity gradient of tidal creeks and other estuaries (Tzortziou et al., 2008; Asmala et al., 2012; Osburn et al., 2012; Letourneau et al., 2021; Logozzo et al., 2021). High $a_{\text{CDOM}}(440)$ and DOC recorded during the late winter and early spring months coincided with high excess rainfall and high flow from the Cape Fear River. During periods of DOC import into the marsh co-occurring with high CFR discharge, terrigenous DOC from the forested wetlands in the Cape Fear River watershed may be transported into BHC (Avery Jr et al., 2003; Avery Jr et al., 2004; Hackney et al., 2007). Other studies report that terrigenous DOC in the Neuse River Estuary, just north of the Cape Fear River Estuary, also were correlated with seasonal patterns of discharge (Peierls and Paerl, 2010; Dixon et al., 2014; Osburn et al., 2016). Likewise, DOC in the Skidaway River Estuary, a tidal salt marsh-dominated estuary in coastal Georgia, peaked synchronously with pulses of freshwater discharge in March 2013 (Bittar et al., 2016). Letourneau and Medeiros (2019) also showed that variations in discharge from the Altamaha River, also in Georgia, was the main driver controlling changes in DOC. Another study in the Neponset salt marsh, Massachusetts, showed that freshwater discharge, especially from episodic events, dominated the total DOM export by rapidly flushing the marsh (Schiebel et al., 2018). These results are particularly important because they show that in addition to the tidal forces governing hydrology in the creek, river discharge acts as a secondary critical source of water flooding the marsh platform and leading to increased mobilization of marsh DOC. Our result for BHC reinforces the importance of hydrology in governing DOC dynamics in salt marshes on the Atlantic and Pacific coasts of the United States (Bogard et al., 2020).

### 4.2. Lateral DOC flux

Quantifying lateral DOC flux between tidal marshes like BHC and their connected estuaries is critical to improve regional and global carbon budgets and reduce uncertainties in model predictions of carbon cycles in a changing climate (Bauer et al., 2013; Najjar et al., 2018; Ward et al., 2020). An estimated 35 % of total organic carbon input to estuaries along the East Coast of the United States comes from tidal marshes (Herrmann et al., 2015). Yet, such quantification has historically been hard to constrain in tidal marshes because of their high spatial and temporal heterogeneity with respect to hydrology and geomorphology, resulting in large uncertainties in DOC fluxes across these terrestrial-aquatic interfaces (Tobias and Neubauer, 2019; Cai, 2011; Wang et al., 2016; Windham-Myers et al., 2018; Ward et al., 2020). However, coupling field-based monitoring of DOC with a numerical hydrodynamic model like SCHISM in this study allowed for a synoptic representation of DOC fluxes in BHC across scales not feasible with field surveys alone. Additionally, the use of high-resolution bathymetry was critical in the development of the high-resolution unstructured mesh grid for use in SCHISM. This grid allowed for a better representation of a system by constraining the boundaries of a tidal marsh and its creek than these prior approaches.

The DOC flux within the marsh-creek interface was seasonally variable. A peak in export in late winter was synchronous with excess rainfall immediately followed by high river discharge, while in early spring, the marsh exhibited a net import during low excess rainfall and river discharge. Such seasonal variability, with higher export in late winter, was also observed in marsh-dominated Bly Creek, North Inlet, South Carolina (Williams et al., 1992), Chesapeake Bay (Tzortziou et al., 2008; Clark et al., 2018; Signorini et al., 2019) and Delaware Bay (Signorini et al., 2019). During high river discharge, the subsequent increased volume of water results in increased water flux that floods the marsh platform, infiltrating into the marsh subsurface and groundwater. During this time of water storage, DOC is extracted from the marsh sediments and plant leachates are flushed out. Once the infiltration capacity of the marsh is exceeded, these processes lead to greater mobilization and export of DOC to the adjacent estuary above any tidal flux (Schiebel et al., 2018).

Porewater sources of DOC may be the key store of carbon flushed out during these events. Wolaver and Spurrier (1988) showed that rainstorms in a South Carolina tidal creek can increase DOC export from the marsh platform through runoff and seepage by disturbing the sediment substrate with a commensurate release of interstitial DOC or additional runoff from near the marsh-upland border caused by elevated groundwater levels. Additionally, Fig. 6 shows that, even in late winter and early spring, episodic, extreme rainfall events can overwhelm the hydrologic storage capacity of tidally-influenced systems, leading to an export of DOC to adjacent coastal systems, similar to fluvial environments (Raymond et al., 2016).

These examples are of particular importance in the context of climate change, where extreme weather events are causing greater impacts on coastal carbon cycling (Paerl et al., 2020). Long-term rainfall records for coastal North Carolina indicate a trend toward increasingly high precipitation associated with tropical cyclones with major implications for biogeochemical cycling in receiving estuary and adjacent coastal waters (Paerl et al., 2020). In North Carolina, extreme precipitation originates mostly from tropical cyclones (Lau et al., 2008; Easterling et al., 2017; Collins et al., 2019). However, our study shows that episodic wintertime precipitation could have significant impacts on carbon stored in coastal ecosystems. Future work could assess dissolved carbon and nitrogen ratios and stable isotope ratios to distinguish between terrestrial and marine sources of organic matter (Leng and Lewis, 2017).

However, dry events can also have important impacts on coastal carbon cycles via lateral flux *into* a marsh. During the early spring from March to April, a relatively dry period, the DOC flux decreased to negative values, which meant an uptake of water and DOC by the marsh. During this period of alternating dry-wet cycles, some DOC could be processed within the marsh, leading to increased vertical fluxes of CO_2_ (Li et al., 2020). During the winter, the resultant DOC flux from BHC mimicked hydrological flow from the Cape Fear River because of increased excess rainfall, showing that in addition to astronomical tides, winter precipitation and river flooding can be key hydrodynamic events creating large exports of carbon from tidal marshes. During the spring season that followed, where excess rainfall averaged 0.3 mm, synchronous with low runoff and a drier period, the marsh imported carbon.

The average daily DOC import of −13.6 ± 6.0 g C m^−2^ d^−1^ was greater than the average daily DOC export of 9.4 ± 8.3 g C m^−2^ d^−1^ during the study period, showing that despite high river flow, the surrounding marsh acted as a sink during this period of low precipitation, resulting in uptake of DOC. Results also revealed the major role that rainfall plays in mobilizing and exporting large quantities of DOC from the exposed marsh surface, consistent with other studies. Notably, the dry periods where DOC uptake by marshes occurs could increase salinity, due to low freshwater inputs, and thus DOC decomposition to CO_2_, with its eventual release from the marsh (Kirwan and Blum, 2011; Qu et al., 2019). Krauss et al. (2018) and Weston (2014) observed that increased salinity influenced microbial processes, specifically selecting for salt-tolerant microbes that drive higher rates of DOC decomposition, leading to the potential for enhanced CO_2_ emissions.

### 4.3. SCHISM model performance assessment

Assessment of model performance showed that SCHISM captured the observed trend in water level and current velocity reasonably well, exhibiting little bias and minimized RMSE, indicative of the model’s ability to simulate the hydrodynamic variability in this tidal creek. Previous studies simulating estuarine water level reported RMSE ranging from 0.12 m to 0.64 m and bias ranging from −0.2 m and 0.15 m (Marsooli and Lin, 2018; Bakhtyar et al., 2020). Current velocities were previously simulated with RMSE ranging from 0.01 to 0.05 ms^−1^ (Jin et al., 2000), highlighting that our SCHISM model performance (RMSE = 0.10 ms^−1^) was within the range of previous model performance. Numerical hydrodynamic models have the advantage of mimicking water bodies with a relatively high spatial and temporal resolution (Edelvang et al., 2005). Additionally, numerical hydrodynamic models have been shown to be an efficient tool to reproduce seasonality observed in coastal and estuarine systems (Leo et al., 2019). The successful use of numerical models as a relatively inexpensive tool for accurately quantifying organic carbon flux between marshes and estuaries was also demonstrated in the Rhode River, Maryland, USA, a subestuary of the Chesapeake Bay (Clark et al., 2020). Our comparison of modeled and observed current velocity direction via the dot product method showed that both datasets disagreed at low and high tide when the tide was changing directions. At low tide, water level is at a minimum with halted flow and velocity while at high tide, water level is at a maximum, with halted flow and velocity (Cummings et al., 2015). This consistent tidal asymmetry in which velocity and flow temporarily flatten at zero movement was also shown in Groves Creek salt marsh, Georgia, USA (Blanton et al., 2010; Sullivan et al., 2015; Codden et al., 2022).

In its current configuration, SCHISM can sufficiently capture the hydrodynamics of BHC, and the reasonable model performance metrics combined with the strong CDOM-to-DOC relationship gives confidence in the predicted DOC flux. Implementation of the numerical model requires sufficient data for validation, which we were able to achieve by collecting high-frequency data over a month. When working in a system like BHC where tidal forces are important, the sampling frequency must be able to resolve tidal effects that are the predominant driver of hydrodynamic variability (Clark et al., 2020). Overall, our results demonstrated that this combined approach using numerical model data and *in-situ* CDOM absorption can provide reasonable estimates of DOC fluxes. Ultimately, merging this approach with data derived from high-spatial resolution satellite platforms would be the next avenue to explore for detection of finer marsh-estuarine biogeochemical fluxes at a global scale and eventually to improve predictions of the role of blue carbon ecosystems like tidal marshes in coastal and global carbon budgets (Cao and Tzortziou, 2021; Tuzcu Kokal et al., 2024).

### 4.4. Future considerations

Globally, tidal marshes only cover approximately 45,000 km^2^, an area slightly larger than the country of Denmark, but account for approximately 30 % of the total C buried in ocean sediments (Duarte et al., 2005; Wang et al., 2021). However, tidal marshes are being lost at a rate of 1–2 % yearly and 25 % of their historical global coverage has been lost (Duarte et al., 2008; Nelleman et al., 2009). The loss of these carbon sinks not only represents a threat to biodiversity and coastal protection but also affects the capacity of the biosphere to remove anthropogenic CO_2_ emissions (Nelleman et al., 2009; Thorne et al., 2015; Fitzgerald and Hughes, 2019). Tidal marshes are vulnerable to both sea level rise and more intense and frequent tropical cyclones (Thorne et al., 2015; Fitzgerald and Hughes, 2019). Sea level rise will likely cause increased rates of inundation, which could increase the mobilization and export of DOC from marshes to estuaries (Arellano et al., 2019; Chambers et al., 2014). However, drought may effectively offset the carbon sequestration capacity of tidal marshes, because, as we discovered in this study, low precipitation may lead to uptake of water and DOC in marshes with the potential for DOC degradation to CO_2_. Future work on climate impacts and anthropogenic disturbances to coastal marshes following the approach presented in this study could be strengthened by incorporating remotely-sensed imagery for a global-scale analysis. This would help inform the extent of diminished carbon storage in tidal marshes and a better understanding on how to manage them in a way to minimize carbon loss and maximize carbon sequestration (Macreadie et al., 2017, 2019; Kelleway et al., 2020).

Future work should focus on expanding this analysis across time and space. Across time, this study only considered the months of January through April in a single year. Additional years of data would be required to establish patterns in seasonality, and considering additional months can help further elucidate drivers of DOC flux in tidal marshes. Increased temporal coverage would also allow for inter- and intra-annual assessments. Across space, a single study site in Bald Head Creek was considered. Assessment of additional marsh ecosystems in the same region may reveal spatial variability in DOC flux and may aid in further constraining coastal carbon stocks.

## 5. Conclusion

Integrating field-based monitoring with numerical modeling holds strong potential for future quantification and monitoring of carbon pools in tidal marshes. We used a linear relationship between CDOM absorption and DOC concentration with the water flux from the SCHISM numerical model to map the spatial variability in DOC and reproduce the hydrodynamics of Bald Head Creek, which allowed us to quantify daily DOC fluxes between the creek and the adjacent Cape Fear River estuary during the winter and spring season. The spatial and temporal scale of our work combining field measurements and numerical modeling would not be possible with field surveys alone.

Overall, late winter-early spring represented a time of net DOC import to the marsh from the adjacent Cape Fear River estuary, although variations in DOC concentration and flux revealed seasonal and tidal dependencies over the study period. When considering drivers of this variability, our study indicates that rainfall events in late winter-early spring drive largescale mobilization and export of DOC to adjacent coastal systems over relatively short time periods, due to the combined impacts of localized precipitation and increased river discharge.

## Supplementary Material

Supplement1

Appendix A. Supplementary data

Supplementary data to this article can be found online at https://doi.org/10.1016/j.ecss.2025.109361.

## Figures and Tables

**Table 1:** Dates of BHIC sampling for quantifying CDOM.

Date	Sampling time (EST)	Tide level at time of sampling (m)	Average 10-day Cape Fear River discharge ($m^{3}s^{-1}$)
6-Jan-21	13:30	0.37	570
13-Jan-21	14:00	1.83	634
27-Jan-21	13:15	1.56	174
3-Feb-21	14:26	0.40	358
10-Feb-21	13:32	1.69	531
17-Feb-21	13:17	0.32	484
24-Feb-21	14:20	1.30	729
3-Mar-21	13:41	0.69	636
10-Mar-21	13:16	1.47	534
17-Mar-21	14:01	0.99	373
24-Mar-21	13:55	0.82	285
7-Apr-21	13:14	1.11	354
14-Apr-21	13:45	1.31	194
21-Apr-21	13:53	0.48	136

**Table 2:** Distribution of each input variable considered in the SCHISM model. Bathymetry and water level are from Bald Head Island, and river discharge is from the Cape Fear River, all of which were used as open boundary conditions for the SCHISM model.

Input variable	Minimum	Maximum	Mean	Median	Standard deviation
Bathymetry (m)	−7.00	5.00	0.23	0.00	0.79
Water level (m)	0.00	0.89	0.34	0.34	0.19
River discharge ($m^{3}/s$)	−906.14	−27.41	−176.50	−75.32	199.22

**Table 3:** Summary of daily *in situ* $a_{\text{CDOM}}(440)$ and DOC values extracted from 14 samples collected in Bald Head Creek between January to April 2021. DOC flux values were generated from the DOC measurements and model-derived current velocity.

Date	$a_{\text{CDOM}}(440)\left(m^{-1}\right)$	DOC ($\text{mg}L^{-1}$)	DOC flux ($g\;C\;m^{-2}{\;d}^{-1}$)	Monthly average DOC flux ($g\;C\;m^{-2}{\;d}^{-1}$)
6-Jan-21	1.40	3.33	−10.19	−17.3 ± 6.8
13-Jan-21	4.46	7.76	−23.77	
27-Jan-21	3.13	5.84	−17.89	
3-Feb-21	1.09	2.88	6.65	14.0 ± 9.9
10-Feb-21	2.91	5.52	12.77	
17-Feb-21	1.51	3.49	8.08	
24-Feb-21	7.56	12.26	28.36	
3-Mar-21	2.19	4.48	3.96	4.84 ± 2.43
10-Mar-21	5.71	9.58	8.46	
17-Mar-21	1.85	3.99	3.52	
24-Mar-21	1.77	3.87	3.42	
7-Apr-21	2.51	4.94	−12.04	−9.94 ± 2.10
14-Apr-21	1.92	4.08	−9.95	
21-Apr-21	1.32	3.22	−7.83	

## Data Availability

I have attached my data as a spreadsheet.

## References

[R1] Altieri AH, Gedan KB, 2015. Climate change and dead zones. Glob. Change Biol 21 (4), 1395–1406. 10.1111/gcb.12754 25385668

[R2] Arellano AR, Bianchi TS, Osburn CL, D’Sa EJ, Ward ND, Oviedo-Vargas D, Joshi ID, Ko DS, Shields MR, Kurian G, 2019. Mechanisms of organic matter export in estuaries with contrasting carbon sources. J. Geophys. Res.: Biogeosciences 124 (10), 3168–3188.

[R3] Asmala E, Stedmon CA, Thomas DN, 2012. Linking CDOM spectral absorption to dissolved organic carbon concentrations and loadings in boreal estuaries. Estuar. Coast Shelf Sci 111, 107–117.

[R4] Avery GB Jr, Willey JD, Kieber RJ, Shank GC, Whitehead RF, 2003. Flux and bioavailability of Cape Fear River and rainwater dissolved organic carbon to Long Bay, southeastern United States. Glob. Biogeochem. Cycles 17 (2).

[R5] Avery GB Jr, Kieber RJ, Willey JD, Shank GC, Whitehead RF, 2004. Impact of hurricanes on the flux of rainwater and cape fear river water dissolved organic carbon to long bay, southeastern United States. Glob. Biogeochem. Cycles 18 (3).

[R6] Bakhtyar R, Maitaria K, Velissariou P, Trimble B, Mashriqui H, Moghimi S, Abdolali A, Van der Westhuysen AJ, Ma Z, Clark EP, 2020. A new 1D/2D coupled modeling approach for a riverine-estuarine system under storm events: application to Delaware River basin. J. Geophys. Res.: Oceans 125 (9), e2019JC015822.

[R7] Bauer JE, Cai W, Raymond PA, Bianchi TS, Hopkinson CS, Regnier PA, 2013. The changing carbon cycle of the coastal ocean. Nature 504 (7478), 61–70. 24305149 10.1038/nature12857

[R8] Becker ML, Luettich RA Jr, Mallin MA, 2010. Hydrodynamic behavior of the cape fear river and estuarine system: a synthesis and observational investigation of discharge-salinity intrusion relationships. Estuar. Coast Shelf Sci 88 (3), 407–418.

[R9] Beckett LH, Baldwin AH, Kearney MS, 2016. Tidal marshes across a Chesapeake Bay subestuary are not keeping up with sea-level rise. PLoS One 11 (7), e0159753. 27467784 10.1371/journal.pone.0159753 PMC4965100

[R10] Benway H, Alin S, Boyer E, Cai WJ, Coble P, Cross J, Friedrichs M, Goñi M, Griffith P, Herrmann M, 2016. No title. A Science Plan for Carbon Cycle Research in North American Coastal Waters.Report of the Coastal CARbon Synthesis (CCARS) Community Workshop. August 19–21, 2014.

[R11] Bittar TB, Berger SA, Birsa LM, Walters TL, Thompson ME, Spencer RG, Mann EL, Stubbins A, Frischer ME, Brandes JA, 2016. Seasonal dynamics of dissolved, particulate and microbial components of a tidal saltmarsh-dominated estuary under contrasting levels of freshwater discharge. Estuar. Coast Shelf Sci 182, 72–85.

[R12] Blanton JO, Garrett AJ, Bollinger JS, Hayes DW, Koffman LD, Amft J, Moore T, 2010. Transport and retention of a conservative tracer in an isolated creek-marsh system. Estuar. Coast Shelf Sci 87 (2), 333–345.

[R13] Bogard MJ, Bergamaschi BA, Butman DE, Anderson F, Knox SH, Windham-Myers L, 2020. Hydrologic export is a major component of coastal wetland carbon budgets. Glob. Biogeochem. Cycles 34 (8), e2019GB006430.

[R14] Boschker HTS, De Brouwer JFC, Cappenberg TE, 1999. The contribution of macrophyte-derived organic matter to microbial biomass in salt-marsh sediments: stable carbon isotope analysis of microbial biomarkers. Limnol. Oceanogr 44 (2), 309–319.

[R15] Bu N, Qu J, Li G, Zhao B, Zhang R, Fang C, 2015. Reclamation of coastal salt marshes promoted carbon loss from previously-sequestered soil carbon pool. Ecol. Eng 81, 335–339.

[R16] Cai W, 2011. Estuarine and coastal ocean carbon paradox: CO2 sinks or sites of terrestrial carbon incineration? Ann. Rev. Mar. Sci 3, 123–145. 10.1146/annurev-marine-120709-142723 21329201

[R17] Chen J, Zhu W, Tian YQ, Yu Q, 2020. Monitoring dissolved organic carbon by combining landsat-8 and sentinel-2 satellites: case study in saginaw River estuary, lake huron. Sci. Total Environ 718, 137374. 32092524 10.1016/j.scitotenv.2020.137374

[R18] Cao F, Tzortziou M, Hu C, Mannino A, Fichot CG, Del Vecchio R, Najjar RG, Novak M, 2018. Remote sensing retrievals of colored dissolved organic matter and dissolved organic carbon dynamics in North American estuaries and their margins. Rem. Sens. Environ 205, 151–165.

[R19] Cao F, Tzortziou M, 2021. Capturing dissolved organic carbon dynamics with Landsat-8 and Sentinel-2 in tidally influenced wetland-estuarine systems. Sci. Total Environ 777, 145910.

[R20] Caretti ON, Bohnenstiehl DR, Eggleston DB, 2021. Spatiotemporal variability in sedimentation drives habitat loss on restored subtidal oyster reefs. Estuaries Coasts 44, 2100–2117. 10.1007/s12237-021-00921-6.

[R21] Chambers LG, Davis SE, Troxler T, Boyer JN, Downey-Wall A, Scinto LJ, 2014. Biogeochemical effects of simulated sea level rise on carbon loss in an Everglades mangrove peat soil. Hydrobiologia 726, 195–211.

[R22] Clark JB, Long W, Hood RR, 2020. A comprehensive estuarine dissolved organic carbon budget using an enhanced biogeochemical model. J. Geophys. Res.: Biogeosciences 125 (5), e2019JG005442.

[R23] Clark JB, Long W, Tzortziou M, Neale PJ, Hood RR, 2018. Wind-driven dissolved organic matter dynamics in a Chesapeake Bay tidal marsh-estuary system. Estuaries Coasts 41 (3), 708–723.

[R24] Coble PG, 2007. Marine optical biogeochemistry: the chemistry of ocean color. Chem. Rev 107 (2), 402–418. 17256912 10.1021/cr050350+

[R25] Codden CJ, Edwards CR, Stubbins A, 2022. Non-conservative behavior of dissolved organic carbon in a Georgia salt marsh creek indicates summer outwelling. Estuar. Coast Shelf Sci 265, 107709.

[R26] Colombano DD, Litvin SY, Ziegler SL, Alford SB, Baker R, Barbeau MA, Cebrián J, Connolly RM, Currin CA, Deegan LA, 2021. Climate change implications for tidal marshes and food web linkages to estuarine and coastal nekton. Estuaries Coasts 44 (6), 1637–1648.

[R27] Collins SM, Yuan S, Tan PN, Oliver SK, Lapierre JF, Cheruvelil KS, Fergus CE, Skaff NK, Stachelek J, Wagner T, Soranno PA, 2019. Winter precipitation and summer temperature predict lake water quality at macroscales. Water Resour. Res 55 (4), 2708–2721.

[R28] Crosby SC, Sax DF, Palmer ME, Booth HS, Deegan LA, Bertness MD, Leslie HM, 2016. Salt marsh persistence is threatened by predicted sea-level rise. Estuar. Coast Shelf Sci 181, 93–99.

[R29] Cummings D, Dalrymple R, Choi K, Jin J, 2015. The Tide-Dominated Han River Delta, Korea: Geomorphology, Sedimentology, and Stratigraphic Architecture. Elsevier.

[R30] Dangendorf S, Hay C, Calafat FM, Marcos M, Piecuch CG, Berk K, Jensen J, 2019. Persistent acceleration in global sea-level rise since the 1960s. Nat. Clim. Change 9 (9), 705–710.

[R31] Deegan LA, Johnson DS, Warren RS, Peterson BJ, Fleeger JW, Fagherazzi S, Wollheim WM, 2012. Coastal eutrophication as a driver of salt marsh loss. Nature 490 (7420), 388–392. 23075989 10.1038/nature11533

[R32] Del Vecchio R, Subramaniam A, 2004. Influence of the amazon river on the surface optical properties of the western tropical North Atlantic Ocean. J. Geophys. Res.: Oceans 109 (C11).

[R33] Dixon JL, Osburn CL, Paerl HW, Peierls BL, 2014. Seasonal changes in estuarine dissolved organic matter due to variable flushing time and wind-driven mixing events. Estuar. Coast Shelf Sci 151, 210–220.

[R34] Doody JP, 2013. Coastal squeeze and managed realignment in southeast England, does it tell us anything about the future? Ocean Coast Manag. 79, 34–41.

[R35] Downing BD, Boss E, Bergamaschi BA, Fleck JA, Lionberger MA, Ganju NK, Schoellhamer DH, Fujii R, 2009. Quantifying fluxes and characterizing compositional changes of dissolved organic matter in aquatic systems in situ using combined acoustic and optical measurements. Limnol Oceanogr. Methods 7 (1), 119–131.

[R36] Duarte CM, Dennison WC, Orth RJ, Carruthers TJ, 2008. The charisma of coastal ecosystems: addressing the imbalance. Estuaries Coasts 31 (2), 233–238.

[R37] Duarte CM, Middelburg JJ, Caraco N, 2005. Major role of marine vegetation on the oceanic carbon cycle. Biogeosciences 2 (1), 1–8.

[R38] Easterling DR, Arnold JR, Knutson T, Kunkel KE, LeGrande AN, Leung LR, Vose RS, Waliser DE, Wehner MF, 2017. Precipitation Change in the United States.

[R39] Edelvang K, Kaas H, Erichsen AC, Alvarez-Berastegui D, Bundgaard K, Jørgensen PV, 2005. Numerical modelling of phytoplankton biomass in coastal waters. J. Mar. Syst 57 (1–2), 13–29.

[R40] Fagherazzi S, Wiberg PL, Temmerman S, Struyf E, Zhao Y, Raymond PA, 2013. Fluxes of water, sediments, and biogeochemical compounds in salt marshes. Ecol. Process 2 (1), 1–16.

[R41] Fichot CG, Benner R, 2012. The spectral slope coefficient of chromophoric dissolved organic matter (S275–295) as a tracer of terrigenous dissolved organic carbon in river-influenced ocean margins. Limnol. Oceanogr 57 (5), 1453–1466.

[R42] FitzGerald DM, Hughes Z, 2019. Marsh processes and their response to climate change and sea-level rise. Annu. Rev. Earth Planet Sci 47, 481–517.

[R43] Fogel ML, Sprague EK, Gize AP, Frey RW, 1989. Diagenesis of organic matter in Georgia salt marshes. Estuar. Coast Shelf Sci 28 (2), 211–230.

[R44] Ganju NK, Schoellhamer DH, Bergamaschi BA, 2005. Suspended sediment fluxes in a tidal wetland: measurement, controlling factors, and error analysis. Estuaries 28 (6), 812–822.

[R45] Gilby BL, Weinstein MP, Baker R, Cebrian J, Alford SB, Chelsky A, Colombano D, Connolly RM, Currin CA, Feller IC, 2021. Human actions alter tidal marsh seascapes and the provision of ecosystem services. Estuaries Coasts 44 (6), 1628–1636.

[R46] Greenberg R, Maldonado JE, Droege S, McDonald MV, 2006. Tidal marshes: a global perspective on the evolution and conservation of their terrestrial vertebrates. Bioscience 56 (8), 675–685.

[R47] Hackney CT, Avery GB, Leonard LA, Posey M, Alphin T, 2007. Biological, chemical, and physical characteristics of tidal freshwater swamp forests of the lower Cape Fear River/Estuary, North Carolina. Ecology of Tidal Freshwater Forested Wetlands of the Southeastern United States. Springer, pp. 183–221.

[R48] Hedges JI, 1992. Global biogeochemical cycles: progress and problems. Mar. Chem 39 (1–3), 67–93.

[R49] Herrmann M, Najjar RG, Kemp WM, Alexander RB, Boyer EW, Cai W, Griffith PC, Kroeger KD, McCallister SL, Smith RA, 2015. Net ecosystem production and organic carbon balance of U.S. East Coast estuaries: a synthesis approach. Glob. Biogeochem. Cycles 29 (1), 96–111.

[R50] Himes-Cornell A, Pendleton L, Atiyah P, 2018. Valuing ecosystem services from blue forests: a systematic review of the valuation of salt marshes, sea grass beds and mangrove forests. Ecosyst. Serv 30, 36–48.

[R51] Holmquist JR, Brown LN, MacDonald GM, 2021. Localized scenarios and latitudinal patterns of vertical and lateral resilience of tidal marshes to sea-level rise in the contiguous United States. Earths Future 9 (6), e2020EF001804.

[R52] Hooker SB, Matsuoka A, Kudela RM, Yamashita Y, Suzuki K, Houskeeper HF, 2020. A global end-member approach to derive a CDOM (440) from near-surface optical measurements. Biogeosciences 17 (2), 475–497.

[R53] Houskeeper HF, Hooker SB, Kudela RM, 2021. Spectral range within global aCDOM (440) algorithms for oceanic, coastal, and inland waters with application to airborne measurements. Rem. Sens. Environ 253, 112155.

[R54] Jiao N, Herndl GJ, Hansell DA, Benner R, Kattner G, Wilhelm SW, Kirchman DL, Weinbauer MG, Luo T, Chen F, Azam F, 2010. Microbial production of recalcitrant dissolved organic matter: long-term carbon storage in the global ocean. Nat. Rev. Microbiol 8, 593–599. 20601964 10.1038/nrmicro2386

[R55] Jiménez-Ramos R, Egea LG, D’Agostino VC, Degrati M, Loizaga R, 2025. Carbon and nitrogen stocks in sediment at Península Valdés Biosphere Reserve: novel insights into the potential contribution of large marine vertebrates to carbon sequestration. Front. Mar. Sci 12, 1500594.

[R56] Jin K, Hamrick JH, Tisdale T, 2000. Application of three-dimensional hydrodynamic model for Lake Okeechobee. J. Hydraul. Eng 126 (10), 758–771.

[R57] Joshi ID, D’Sa EJ, Osburn CL, Bianchi TS, Ko DS, Oviedo-Vargas D, Arellano AR, Ward ND, 2017. Assessing chromophoric dissolved organic matter (CDOM) distribution, stocks, and fluxes in Apalachicola Bay using combined field, VIIRS ocean color, and model observations. Rem. Sens. Environ 191, 359–372.

[R58] Kelleway JJ, Serrano O, Baldock JA, Burgess R, Cannard T, Lavery PS, Lovelock CE, Macreadie PI, Masque P, Newnham M, 2020. A national approach to greenhouse gas abatement through blue carbon management. Glob. Environ. Change 63, 102083.

[R59] Kirwan ML, Blum LK, 2011. Enhanced decomposition offsets enhanced productivity and soil carbon accumulation in coastal wetlands responding to climate change. Biogeosciences 8 (4), 987–993.

[R60] Knobloch AL, Reay WG, Canuel EA, 2021. Carbon pools differ in source and temporal patterns in a tidal marsh creek system of the York River, VA estuary. Estuaries Coasts 1–18.

[R61] Krauss KW, Noe GB, Duberstein JA, Conner WH, Stagg CL, Cormier N, Jones MC, Bernhardt CE, Graeme Lockaby B, From AS, Doyle TW, Day RH, Ensign SH, Pierfelice KN, Hupp CR, Chow AT, Whitbeck JL, 2018. The role of the upper tidal estuary in wetland blue carbon storage and flux. Glob. Biogeochem. Cycles 32 (5), 817–839.

[R62] Lalonde K, Middlestead P, Gélinas Y, 2014. Automation of ^13^C/^12^C ratio measurement for freshwater and seawater DOC using high temperature combustion. Limnol Oceanogr. Methods 12 (12), 816–829.

[R63] Lau KM, Zhou YP, Wu HT, 2008. Have tropical cyclones been feeding more extreme rainfall? J. Geophys. Res. Atmos 113 (D23).

[R64] Lehrter JC, Ko DS, Murrell MC, Hagy JD, Schaeffer BA, Greene RM, Gould RW, Penta B, 2013. Nutrient distributions, transports, and budgets on the inner margin of a river-dominated continental shelf. J. Geophys. Res.: Oceans 118 (10), 4822–4838.

[R65] Leng MJ, Lewis JP, 2017. C/N ratios and carbon isotope composition of organic matter in estuarine environments. In: Weckström K, Saunders K, Gell P, Skilbeck C (Eds.), Applications of Paleoenvironmental Techniques in Estuarine Studies, vol. 20. Developments in Paleoenvironmental Research, pp. 213–237.

[R66] Leo KL, Gillies CL, Fitzsimons JA, Hale LZ, Beck MW, 2019. Coastal habitat squeeze: a review of adaptation solutions for saltmarsh, mangrove and beach habitats. Ocean Coast Manag. 175, 180–190.

[R67] Letourneau ML, Medeiros PM, 2019. Dissolved organic matter composition in a marsh-dominated estuary: response to seasonal forcing and to the passage of a hurricane. J. Geophys. Res.: Biogeosciences 124 (6), 1545–1559.

[R68] Letourneau ML, Schaefer SC, Chen H, McKenna AM, Alber M, Medeiros PM, 2021. Spatio-temporal changes in dissolved organic matter composition along the salinity gradient of a marsh-influenced estuarine complex. Limnol. Oceanogr 66 (8), 3040–3054.

[R69] Lewis CJE, Baldock JA, Hawke B, Gadd PS, Zawadzki A, Heijnis H, Jacobsen GE, Rogers K, Macreadie PI, 2019. Impacts of land reclamation on tidal marsh ‘blue carbon stocks. Sci. Total Environ 672, 427–437. 30965258 10.1016/j.scitotenv.2019.03.345

[R70] Li J, Qu W, Han G, Lu F, Zhou Y, Song W, Baohua X, Eller F, 2020. Effects of drying-rewetting frequency on vertical and lateral loss of soil organic carbon in a tidal salt marsh. Wetlands 40, 1433–1443.

[R71] Logozzo L, Tzortziou M, Neale P, Clark JB, 2021. Photochemical and microbial degradation of chromophoric dissolved organic matter exported from tidal marshes. J. Geophys. Res.: Biogeosciences 126 (4), e2020JG005744.

[R72] Macreadie PI, Anton A, Raven JA, Beaumont N, Connolly RM, Friess DA, Kelleway JJ, Kennedy H, Kuwae T, Lavery PS, 2019. The future of blue carbon science. Nat. Commun 10 (1), 1–13. 31488846 10.1038/s41467-019-11693-w PMC6728345

[R73] Macreadie PI, Hughes AR, Kimbro DL, 2013. Loss of ‘blue carbon’ from coastal salt marshes following habitat disturbance. PLoS One 8 (7), e69244. 23861964 10.1371/journal.pone.0069244 PMC3704532

[R74] Macreadie PI, Nielsen DA, Kelleway JJ, Atwood TB, Seymour JR, Petrou K, Connolly RM, Thomson AC, Trevathan-Tackett SM, Ralph PJ, 2017. Can we manage coastal ecosystems to sequester more blue carbon? Front. Ecol. Environ 15 (4), 206–213.

[R75] Mannino A, Russ ME, Hooker SB, 2008. Algorithm development and validation for satellite-derived distributions of DOC and CDOM in the US Middle Atlantic Bight. J. Geophys. Res.: Oceans 113 (C7).

[R76] Marsooli R, Lin N, 2018. Numerical modeling of historical storm tides and waves and their interactions along the U.S. East and Gulf coasts. J. Geophys. Res.: Oceans 123 (5), 3844–3874.

[R77] Mason CF, Underwood G, Baker NR, Davey PA, Davidson I, Hanlon A, Long SP, Oxborough K, Paterson DM, Watson A, 2003. The role of herbicides in the erosion of salt marshes in Eastern England. Environ. Pollut 122 (1), 41–49. 12535594 10.1016/s0269-7491(02)00284-1

[R78] Massicotte P, Asmala E, Stedmon C, Markager S, 2017. Global distribution of dissolved organic matter along the aquatic continuum: across rivers, lakes and oceans. Sci. Total Environ 609, 180–191. 28738200 10.1016/j.scitotenv.2017.07.076

[R79] Maxwell TL, Rovai AS, Adame MF, Adams JB, Álvarez-Rogel J, Austin WEN, Beasy K, Boscutti F, Böttcher ME, Bouma TJ, Bulmer RH, Burden A, Burke SA, Camacho S, Chaudhary DR, Chmura GL, Copertino M, Cott GM, Craft C, Day J, de los Santos C, Denis L, Ding W, Ellison JC, Worthington TA, 2023. Global dataset of soil organic carbon intidal marshes. Sci. Data 10 (1), 797. 37952023 10.1038/s41597-023-02633-x PMC10640612

[R80] Mcleod E, Chmura GL, Bouillon S, Salm R, Björk M, Duarte CM, Lovelock CE, Schlesinger WH, Silliman BR, 2011. A blueprint for blue carbon: toward an improved understanding of the role of vegetated coastal habitats in sequestering CO_2_. Front. Ecol. Environ 9 (10), 552–560.

[R81] Menendez A, Tzortziou M, Neale P, Megonigal P, Powers L, Schmitt-Kopplin P, Gonsior M, 2022. Strong dynamics in tidal marsh DOC export in response to natural cycles and episodic events from continuous monitoring. J. Geophys. Res.: Biogeosciences 127 (7), e2022JG006863.

[R82] Mishra SK, Singh V, 2003. Soil Conservation Service Curve Number (SCS-CN) Methodology, vol. 42. Springer Science and Business Media.

[R83] Najjar RG, Herrmann M, Alexander R, Boyer EW, Burdige DJ, Butman D, Cai W, Canuel EA, Chen RF, Friedrichs MA, 2018. Carbon budget of tidal wetlands, estuaries, and shelf waters of eastern North America. Glob. Biogeochem. Cycles 32 (3), 389–416.

[R84] Nelleman C, Corcoran E, Duarte CM, 2009. Blue Carbon. A Rapid Response Assessment.UNEP: GRID-Arendal

[R85] Nerem RS, Beckley BD, Fasullo JT, Hamlington BD, Masters D, Mitchum GT, 2018. Climate-change–driven accelerated sea-level rise detected in the altimeter era. In: Proceedings of the National Academy of Sciences, 115, pp. 2022–2025, 9. 10.1073/pnas.1717312115 PMC5834701 29440401

[R86] Office for Coastal Management, 2018. NOAA Post Hurricane Sandy Topobathymetric LiDAR Mapping for Shoreline Mapping from 2010-06-15 to 2010-08-15. NOAA National Centers for Environmental Information, 2014. https://inport.nmfs.noaa.gov/inport/item/48141.

[R87] Osburn CL, Boyd TJ, Montgomery MT, Bianchi TS, Coffin RB, Paerl HW, 2016. Optical proxies for terrestrial dissolved organic matter in estuaries and coastal waters. Front. Mar. Sci 2, 127.

[R88] Osburn CL, Mikan MP, Etheridge JR, Burchell MR, Birgand F, 2015. Seasonal variation in the quality of dissolved and particulate organic matter exchanged between a salt marsh and its adjacent estuary. J. Geophys. Res.: Biogeosciences 120 (7), 1430–1449.

[R89] Osburn CL, Handsel LT, Mikan MP, Paerl HW, Montgomery MT, 2012. Fluorescence tracking of dissolved and particulate organic matter quality in a river-dominated estuary. Environ. Sci. Technol 46 (16), 8628–8636. 22803700 10.1021/es3007723

[R90] Osburn CL, Morris DP, 2003. Photochemistry of chromophoric dissolved organic matter in natural waters. In: UV Effects in Aquatic Organisms and Ecosystems, 1, pp. 185–217.

[R91] Paerl HW, Hall NS, Hounshell AG, Rossignol KL, Barnard MA, Luettich RA, Rudolph JC, Osburn CL, Bales J, Harding LW, 2020. Recent increases of rainfall and flooding from tropical cyclones (TCs) in North Carolina (USA): implications for organic matter and nutrient cycling in coastal watersheds. Biogeochemistry 150, 197–216.

[R92] Pahlevan N, Chittimalli SK, Balasubramanian SV, Vellucci V, 2019. Sentinel-2/Landsat-8 product consistency and implications for monitoring aquatic systems. Rem. Sens. Environ 220, 19–29.

[R93] Pan X, Mannino A, Russ ME, Hooker SB, 2008. Remote sensing of the absorption coefficients and chlorophyll a concentration in the United States southern middle Atlantic Bight from SeaWiFS and MODIS-Aqua. J. Geophys. Res.: Oceans 113 (C11).

[R94] Peierls BL, Paerl HW, 2010. Temperature, organic matter, and the control of bacterioplankton in the Neuse River and Pamlico Sound estuarine system. Aquat. Microb. Ecol 60 (2), 139–149.

[R95] Qu W, Li J, Han G, Wu H, Song W, Zhang X, 2019. Effect of salinity on the decomposition of soil organic carbon in a tidal wetland. J. Soils Sediments 19, 609–617.

[R96] Raymond PA, Saiers JE, Sobczak WV, 2016. Hydrological and biogeochemical controls on watershed dissolved organic matter transport: pulse-shunt concept. Ecology 97 (1), 5–16. 27008769 10.1890/14-1684.1

[R97] Saintilan N, Kovalenko KE, Guntenspergen G, Rogers K, Lynch JC, Cahoon DR, Lovelock CE, Friess DA, Ashe E, Krauss KW, Cormier N, 2022. Constraints on the adjustment of tidal marshes to accelerating sea level rise. Science 377 (6605), 523–527. 35901146 10.1126/science.abo7872

[R98] Sanger D, Parker C, 2016. Guide to the Salt Marshes and Tidal Creeks of the Southeastern United States. South Carolina Department of Natural Resources.

[R99] Schiebel HN, Gardner GB, Wang X, Peri F, Chen RF, 2018. Seasonal export of dissolved organic matter from a New England salt marsh. J. Coast Res 34 (4), 939–954.

[R100] Sherrill BL, Snider AG, DePerno CS, 2010. White-tailed deer on a barrier island: implications for preserving an ecologically important maritime forest. In: Paper Presented at the Proceedings from the Annual Conference of the Southeast Association of Fish and Wildlife, vol. 64, pp. 38–43 (2010).

[R101] Signorini SR, Mannino A, Friedrichs MA, St-Laurent P, Wilkin J, Tabatabai A, Najjar RG, Hofmann EE, Da F, Tian H, 2019. Estuarine dissolved organic carbon flux from space: with application to Chesapeake and Delaware Bays. J. Geophys. Res.: Oceans 124 (6), 3755–3778.

[R102] Sullivan JC, Torres R, Garrett A, Blanton J, Alexander C, Robinson M, Moore T, Amft J, Hayes D, 2015. Complexity in salt marsh circulation for a semienclosed basin. J. Geophys. Res.: Earth Surf 120 (10), 1973–1989.

[R103] Taylor MD, Baker R, Simenstad C, Weinstein MP, 2021. Concepts and controversies in tidal marsh ecology revisited. Estuaries Coasts 1–4.

[R104] Teal JM, 1962. Energy flow in the salt marsh ecosystem of Georgia. Ecology 43 (4), 614–624.

[R105] Thorne KM, Mattsson BJ, Takekawa J, Cummings J, Crouse D, Block G, Bloom V, Gerhart M, Goldbeck S, Huning B, 2015. Collaborative decision-analytic framework to maximize resilience of tidal marshes to climate change. Ecol. Soc 20 (1).

[R106] Thornton DCO, 2014. Dissolved organic matter (DOM) release by phytoplankton in the contemporary and future ocean. Eur. J. Phycol 49, 20–46.

[R107] Tisserand D, Daval D, Truche L, Fernandez-Martinez A, Sarret G, Spadini L, Nemery J, 2024. Recommendations and good practices for dissolved organic carbon (DOC) analyses at low concentrations. MethodsX 12, 102663. 38559387 10.1016/j.mex.2024.102663 PMC10979086

[R108] Tobias C, Neubauer SC, 2019. Salt marsh biogeochemistry—an overview. Coastal Wetlands 539–596.

[R109] Tuzcu Kokal A, Harringmeyer JP, Cronin-Golomb O, Weiser MW, Hong J, Ghosh N, Swanson J, Zhu X, Musaoglu N, Fichot CG, 2024. Capturing the dynamics of dissolved organic carbon (DOC) in tidal saltmarsh estuaries using remote-sensing-informed models. J. Geophys. Res.: Biogeosciences 129 (10), e2024JG008059.

[R110] Tzortziou M, Neale PJ, Osburn CL, Megonigal JP, Maie N, Jaffé R, 2008. Tidal marshes as a source of optically and chemically distinctive colored dissolved organic matter in the chesapeake bay. Limnol. Oceanogr 53 (1), 148–159.

[R111] Vantrepotte V, Danhiez F, Loisel H, Ouillon S, Mériaux X, Cauvin A, Dessailly D, 2015. CDOM-DOC relationship in contrasted coastal waters: implication for DOC retrieval from ocean color remote sensing observation. Opt. Express 23 (1), 33–54. 25835652 10.1364/OE.23.000033

[R112] Wang F, Sanders CJ, Santos IR, Tang J, Schuerch M, Kirwan ML, Kopp RE, Zhu K, Li X, Yuan J, 2021. Global blue carbon accumulation in tidal wetlands increases with climate change. Natl. Sci. Rev 8 (9), nwaa296. 34691731 10.1093/nsr/nwaa296 PMC8433083

[R113] Wang ZA, Kroeger KD, Ganju NK, Gonneea ME, Chu SN, 2016. Intertidal salt marshes as an important source of inorganic carbon to the coastal ocean. Limnol. Oceanogr 61 (5), 1916–1931.

[R114] Ward ND, Megonigal JP, Bond-Lamberty B, Bailey VL, Butman D, Canuel EA, Diefenderfer H, Ganju NK, Goñi MA, Graham EB, 2020. Representing the function and sensitivity of coastal interfaces in earth system models. Nat. Commun 11 (1), 1–14. 32424260 10.1038/s41467-020-16236-2 PMC7235091

[R115] Watson CS, White NJ, Church JA, King MA, Burgette RJ, Legresy B, 2015. Unabated global mean sea-level rise over the satellite altimeter era. Nat. Clim. Change 5 (6), 565–568.

[R116] Weston NB, 2014. Declining sediments and rising seas: an unfortunate convergence for tidal wetlands. Estuaries Coasts 37 (1), 1–23.

[R117] Weston NB, Neubauer SC, Velinsky DJ, Vile MA, 2014. Net ecosystem carbon exchange and the greenhouse gas balance of tidal marshes along an estuarine salinity gradient. Biogeochemistry 120 (1), 163–189.

[R118] Williams TM, Wolaver TG, Dame RF, Spurrier JD, 1992. The Bly Creek ecosystem study—organic carbon transport within a euhaline salt marsh basin, North Inlet, South Carolina. J. Exp. Mar. Biol. Ecol 163 (1), 125–139.

[R119] Windham-Myers L, Crooks S, Troxler TG, 2018. A Blue Carbon Primer: the State of Coastal Wetland Carbon Science, Practice and Policy. CRC Press.

[R120] Wolaver TG, Spurrier JD, 1988. Carbon transport between a euhaline vegetated marsh in South Carolina and the adjacent tidal creek: contributions via tidal inundation, runoff and seepage. Mar. Ecol. Prog. Ser 53–62.

[R121] Worthington TA, Spalding M, Landis E, Maxwell TL, Navarro A, Smart LS, Murray NJ, 2024. The distribution of global tidal marshes from earth observation data. Global Ecol. Biogeogr 33 (8), e13852.

[R122] Zhang YJ, Ye F, Stanev EV, Grashorn S, 2016. Seamless cross-scale modeling with SCHISM. Ocean Model. 102, 64–81.

